# Factors Affecting Dyslipidemia among Korean Adolescents: An Analysis Using the 8th Korea National Health and Nutrition Examination Survey (2021)

**DOI:** 10.3390/children10101618

**Published:** 2023-09-28

**Authors:** Ji-Hye Choe, Kyung-Sook Bang, Sang-Youn Jang

**Affiliations:** 1 Center for Human-Caring Nurse Leaders for the Future by Brain Korea 21 (BK 21) Four Project, Seoul National University, Seoul 03080, Republic of Korea; jihye427@snu.ac.kr; 2 College of Nursing, The Research Institute of Nursing Science, Seoul National University, Seoul 03080, Republic of Korea; 3 College of Nursing, Seoul National University, Seoul 03080, Republic of Korea; wish3640@snu.ac.kr

**Keywords:** dyslipidemia, adolescents, risk factors, KNHANES

## Abstract

A high prevalence of dyslipidemia has recently been shown not only in adults, but also in adolescents. When occurring in adolescence, dyslipidemia is a risk factor for cardiovascular disease in adulthood. This study aimed to identify significant factors affecting dyslipidemia in South Korean adolescents. We used data from the third year of the 8th Korea National Health and Nutrition Examination Survey (KNHANES VIII-3) on 381 Korean adolescents aged 12–18 years. The data were analyzed using frequency analysis, descriptive statistics, the Rao–Scott χ test, the *t*-test, and univariate and multivariate logistic regression using complex sample analysis. On a weighted population basis, 28.1% of the adolescents among the participants were identified as the group with dyslipidemia. Obesity, waist circumference, serum uric acid, and alanine aminotransferase (ALT) were significantly related to the prevalence of dyslipidemia. Physical activity 4–7 days a week was related to a decrease in the prevalence of dyslipidemia only in male adolescents. The results of this study can be used as evidence for the risk assessment of adolescent dyslipidemia and the establishment of systematic health management guidelines according to risk factors for the prevention of adolescent dyslipidemia.

## 1. Introduction

Dyslipidemia, when it develops during childhood and adolescence, can increase the risk of cardiovascular disease in adulthood. Recently, dyslipidemia has been shown to have a high prevalence not only in adults, but also in adolescents. A study found that from 2007 to 2018, the prevalence of dyslipidemia in Korean adolescents decreased slightly from 31.8% to 28.7% for males and from 28.9% to 28.2% for females [1], but still shows a high prevalence of nearly 30%. Dyslipidemia aggravates atherosclerosis by accompanying insulin resistance and hypertension, which are other risk factors for cardiovascular disease [2]. Cardiovascular disease usually develops after the age of 40, but the accumulation of early atherosclerotic fat glands is known to occur from childhood [3]. In the management of dyslipidemia, not only drug intake but also lifestyle and eating habits play an important role, and the recent increase in obesity rates in adolescents due to unbalanced diets and a lack of exercise [4] affects the incidence of dyslipidemia in the age group.

According to the 2017 Korean clinical practice guidelines for dyslipidemia in children and adolescents based on the guidelines of the National Heart Lung and Blood Institute (NHBLI) in the US, dyslipidemia in adolescents is defined as at least one of a total cholesterol (TC) of 200 mg/dL or higher, triglyceride (TG) of 130 mg/dL or higher (10–19 years), low-density lipoprotein-cholesterol (LDL-C) of 130 mg/dL or higher, high-density lipoprotein-cholesterol (HDL-C) of 40 mg/dL or lower, and non-high-density lipoprotein-cholesterol (non-HDL-C) of 145 mg/dL or higher [5,6]. Obesity [7], short stature [2], and maternal lipid levels [8] were reported as factors related to dyslipidemia in children and adolescents, and hyperuricemia [9] was reported as a factor related to dyslipidemia in adults. The TG/HDL-C ratio and the TC/HDL-C ratio are highly predictive indicators of metabolic syndrome in adolescents [10], and the TG/HDL-C ratio is an indicator of insulin resistance, and related to cardiovascular mortality [11]. Additionally, hemoglobin (Hb) and hematocrit (Hct) increase with obesity and blood pressure increase in children and adolescents [12], and insulin resistance and uric acid are positively correlated in obese adolescents and children [13].

Furthermore, it was found that the higher the ALT level in children and adolescents, the higher the risk of cardiovascular disease related to obesity, high blood pressure, and dyslipidemia [14]. It has also been reported that high ALT levels in adolescents are related to male gender, obesity, older age, and abnormal lipid levels, while high AST levels in adolescents are related to younger age, obesity, and abnormal lipid levels [15]. As such, since dyslipidemia is related to various factors including obesity, continuous research is needed to identify risk factors and to determine the degree to which they affect dyslipidemia. These studies will be an important basis for the development of intervention strategies according to each risk factor for dyslipidemia. However, few studies have identified the influencing factors of dyslipidemia in Korean adolescents based on national survey data. Therefore, it is necessary to analyze the differences between the dyslipidemia group and the normal group in adolescents, as well as to identify the factors affecting dyslipidemia in adolescents.

This study aims to identify significant factors influencing dyslipidemia in Korean adolescents aged 12–18 years using data from the third year of the 8th Korea National Health and Nutrition Examination Survey (KNHANES).

## 2. Materials and Methods

### 2.1. Research Design

This secondary data analysis study was designed to identify factors affecting dyslipidemia among Korean adolescents aged 12–18 years using data from the third year (2021) of the 8th KNHANES.

### 2.2. Data Source and Study Population

The KNHANES is a national statutory survey performed every year to survey the health behavior, prevalence of chronic diseases, and food and nutritional intake among South Koreans over the age of one [16]. It includes health surveys, physical examination surveys, and nutrition surveys, and in this study data from the 8th third year (2021) health surveys and physical examination surveys were used. The KNHANES used the most recent population and housing census data available as a sampling frame [16]. The sample design of the KNHANES used two-stage stratified cluster sampling, and was designed to extract representative samples for the entire target population of Korea by using rolling sampling so that samples from three years within the cycle were similar to each other [16].

In the third year (2021) of the 8th KNHANES, which was the source data for this study, the number of survey subjects was 9682, and 7090 people (73.2%) participated in at least one of the health survey, physical examination survey, and nutrition survey [16]. Among the 7090 survey participants, 442 (6.2%) were adolescents aged 12–18 years. Among 442 adolescents, 381 (201 males and 180 females) were included as the final analysis subjects of this study after excluding those with missing TC, TG, and HDL-C levels in their dyslipidemia blood tests. Among the 381 subjects for final analysis, those who met the diagnostic criteria for dyslipidemia in children and adolescents were selected as the dyslipidemia group. Among the blood tests corresponding to the diagnostic criteria for dyslipidemia, the values of raw data were used for TC, TG, and HDL-C. LDL-C used the value calculated using Friedewald’s formula [17] for subjects with a TG level of less than 400 mg/dL, and none of the subjects analyzed in this study had a TG level of more than 400 mg/dL. For non-HDL-C, the value obtained by subtracting HDL-C from TC was used.

Applying the complex sample analysis method according to the guidelines of the KNHANES, the sample of 381 adolescents in this study represents approximately 3.07 million Korean adolescents. According to previous research, those who take hyperlipidemia and hypertension drugs that can affect serum lipid concentration fall under the exclusion criteria of [10], but none of the subjects in this study took these drugs. In addition, the long-term use of steroids and various accompanying diseases such as hypothyroidism, Cushing disease, liver disease, and renal syndrome can affect lipid levels, but these diseases rarely occur in children and adolescents [8] and none of the subjects in this study had thyroid disease, liver cancer, or kidney disease.

The KNHANES was performed to the approval of the Research Ethics Committee of the Korea Disease Control and Prevention Agency (approval number: 2018-01-03-3C-A), and this study was approved by the institutional review board of the researchers’ affiliated university (IRB No. E2308/001-005). After downloading the raw data that guaranteed anonymity after the researcher agreed to ‘Consent to Collect and Use Personal Information for Users of the KNHANES’ and ‘Pledge to Implement Statistical User Compliance’, an analysis for this study was performed.

### 2.3. Study Variables

#### 2.3.1. Dyslipidemia

The diagnostic criteria for dyslipidemia in children and adolescents are at least one of TC 200 mg/dL or higher, TG 130 mg/dL or higher (10–19 years old), LDL-C 130 mg/dL or higher, HDL-C 40 mg/dL or lower, and non-HDL-C 145 mg/dL or higher [5]. The difference from the diagnostic criteria for adult dyslipidemia is that the standard level for hypertriglyceridemia is defined as more than 150 mg/dL for adults and more than 130 mg/dL for adolescents. In this study, participants with at least one of hypercholesterolemia, hypertriglyceridemia, hyper-LDL-cholesterolemia, hypo-HDL-cholesterolemia, and hyper-non-HDL-cholesterolemia were classified as the adolescent dyslipidemia group (TC ≥ 200 mg/dL or TG ≥ 130 mg/dL or LDL-C ≥ 130 mg/dL or HDL-C < 40 mg/dL or non-HDL-C ≥ 145 mg/dL), and those who did not fall under any of the five criteria were classified as the normal group.

#### 2.3.2. Demographic, Lifestyle, Psychological, and Family History Variables of Adolescents

Sex, age, and household income were included in the demographic characteristics; smoking experience, drinking experience, and physical activity for at least 60 min a day were included in lifestyle factors; stress perception level was included in the psychological factors; and parents’ diagnosis of hyperlipidemia was included in family history. These factors were divided into two groups: a normal group and a group with dyslipidemia.

The subjects were divided into two age groups: ‘12–14 years old’ and ‘15–18 years old’. Household income was classified into the quartiles of ‘low’, ‘middle low’, ‘middle high’, and ‘high’ based on the value obtained by dividing monthly household income by the square root of the number of household members according to the response to the survey. Smoking experience was divided into two groups, ‘no’ and ‘yes’, according to the response to the experience of smoking, and drinking experience was divided into two groups, ‘no’ and ‘yes’, according to the response to the lifetime drinking experience. Based on the responses to the survey, the number of days in which physical activities was classified into three groups: ‘none in the last 7 days’, ‘1–3 days a week’, and ‘4–7 days a week’. The stress perception level was classified into three groups, ‘much’, ‘a little’, and ‘hardly’, based on the response to the question about how much stress you feel in your daily life. Parents’ diagnosis of hyperlipidemia was classified into three groups, ‘no’, ‘yes’, and ‘don’t know or no answer’, according to the doctor’s diagnosis and other responses.

#### 2.3.3. Physiological and Biochemical Variables of Adolescents

Physiological and biochemical indicator variables were also analyzed through the classification into normal and dyslipidemia groups. Variables of physiological indicators included body mass index (BMI) percentile, height, waist circumference, systolic blood pressure (SBP), and diastolic blood pressure (DBP). The classification of underweight is given if the BMI percentile is less than the 5th percentile, the classification of normal if it is more than the 5th percentile and less than the 85th percentile, overweight given if it is more than the 85th percentile and less than the 95th percentile, and obesity if it is more than the 95th percentile [18]. SBP and DBP were measured using a mercury-free blood pressure meter on the right arm three times after resting for 5 min in a sitting position, and the final blood pressure used in the analysis was the average of the second and third measurements.

Variables of biochemical indicators include total cholesterol (TC), triglyceride (TG), low-density lipoprotein-cholesterol (LDL-C), high-density lipoprotein-cholesterol (HDL-C), non-high-density lipoprotein-cholesterol (non-HDL-C), the ratio of triglycerides to high-density lipoprotein-cholesterol (TG/HDL-C), the ratio of total cholesterol to high-density lipoprotein-cholesterol (TC/HDL-C), hemoglobin (Hb), hematocrit (Hct), fasting plasma glucose (FPG), hemoglobin A1C (HbA1C), serum uric acid, aspartate aminotransferase (AST), and alanine aminotransferase (ALT). Blood collection was performed through venous puncture after maintaining a fasting time for more than 8 h. LDL-C levels were calculated using Friedewald’s formula (LDL = TC − HDL − TG/5) [17]. Non-HDL-C levels were calculated by subtracting the HDL-C level from the TC level. The ratio of TG to HDL-C was calculated by dividing the TG level by the HDL-C level. The ratio of TC to HDL-C was calculated by dividing the TC level by the HDL-C level.

### 2.4. Data Analysis

Since the data from the KNHANES were extracted using the two-stage stratified cluster sampling design, the contents of the complex sample design consisting of weights, stratified variables, and cluster variables should be applied during data analysis [16]. In compliance with this, this study applied a complex sample design analysis method when analyzing data, and all data were analyzed through SPSS version 25.0 (IBM Corp., New York, NY, USA).

To compare differences in demographic characteristics, lifestyle and psychological factors, family history, physiological and biochemical indicators of the normal group, and the dyslipidemia group of adolescents, a complex sample analysis method was used to obtain unweighted frequency, and weighted percentage, mean and standard errors, and the Rao–Scott χ^2^ test and the *t*-test were performed. A complex sample univariate and multivariate logistic regression analysis was performed to find significant factors affecting dyslipidemia in adolescents. During the analysis, missing values were treated as valid values, and statistical significance was determined when the *p*-value was less than 0.05.

## 3. Results

### 3.1. Comparison of Demographic, Lifestyle, Psychological and Family History Variables in the Normal Group and the Dyslipidemia Group (n = 381, N = 3,072,013)

The subjects of this study, a total of 381 adolescents aged 12–18, represent 3,072,013 Korean adolescents. Of the 381 subjects, 201 were male and 180 were female. Based on the weighted population, 28.1% of the total subjects were in the dyslipidemia group and 71.9% were in the normal group. The variable that showed a significant difference between the dyslipidemia group and the normal group according to demographic, lifestyle, psychological, and family history characteristics was the mother’s diagnosis of hyperlipidemia (Table 1). The prevalence of dyslipidemia in subjects whose mothers were diagnosed with hyperlipidemia was 100%, which was higher than in subjects whose mothers were not diagnosed with hyperlipidemia (26.6%) (*p* = 0.001).

### 3.2. Differences in Physiological and Biochemical Indicators When Comparing the Normal Group and the Dyslipidemia Group (n = 381, N = 3,072,013)

The variables that showed significant differences between the dyslipidemia group and the normal group according to physiological and biochemical indicators were BMI percentile, waist circumference, serum uric acid, and ALT (Table 2). Obese subjects had a 58.6% prevalence of dyslipidemia, higher than the 37.7% of overweight subjects and 18.8% of underweight and normal subjects (*p* < 0.001). Looking at the variables corresponding to the diagnostic criteria for dyslipidemia, the averages of TC, TG, LDL-C, and non-HDL-C levels were significantly higher in the dyslipidemia group than in the normal group (*p* < 0.001), and the average of HDL-C levels was significantly lower (*p* < 0.001). In addition, the average TG/HDL-C ratio and TC/HDL-C ratio were significantly higher in the dyslipidemia group than in the normal group (*p* < 0.001).

### 3.3. Factors Affecting Dyslipidemia among Korean Male and Female Adolescents (n = 381, N = 3,072,013)

A complex sample univariate logistic regression analysis was performed to identify the factors affecting dyslipidemia in male and female adolescents. A total of 381 subjects, 201 male and 180 female adolescents, were included in the analysis.

As a result of the analysis, obesity, waist circumference, serum uric acid, and ALT were significant factors affecting dyslipidemia in both male and female adolescents (Table 3). If the BMI percentile was in the range for ‘obesity’ compared to ‘underweight and normal’, the odds ratio for the prevalence of dyslipidemia was 5.26 times in males and 7.76 times in females. When the waist circumference increased by 1 unit, the odds ratio for the prevalence of dyslipidemia was 1.06 times in males and 1.13 times in females. When the serum uric acid level increased by 1 unit, the odds ratio for the prevalence of dyslipidemia was 1.56 times in males and 2.27 times in females. When the ALT level increased by 1 unit, the odds ratio for the prevalence of dyslipidemia was 1.03 times in males and 1.08 times in females.

Looking at the variables corresponding to the diagnostic criteria for dyslipidemia, when LDL-C and TC levels increased by 1 unit, the odds ratio for the prevalence of dyslipidemia was 1.03 times in males and 1.04 times in females. When the TG level increased by 1 unit, the odds ratio for the prevalence of dyslipidemia was 1.06 times in males and 1.04 times in females. When HDL-C levels increased by 1 unit, the odds ratio for the prevalence of dyslipidemia was 0.88 times in both males and females, and when non-HDL-C levels increased by 1 unit, the odds ratio for the prevalence of dyslipidemia was 1.05 times in males and 1.07 times in females. In addition, when the TG/HDL-C ratio increased by 1 unit, the odds ratio for the prevalence of dyslipidemia was 13.44 times in males and 6.81 times in females, and when the TC/HDL-C ratio increased by 1 unit, the odds ratio for the prevalence of dyslipidemia was 19.91 times in males and 32.61 times in females.

Physical activity 4–7 days a week was a significant factor affecting dyslipidemia only in male adolescents (Table 3). In male adolescents, the odds ratio for the prevalence of dyslipidemia was 0.24 times when the number of days of physical activity was ‘4–7 days a week’, compared to ‘none in the last 7 days’.

In addition, to identify factors affecting dyslipidemia, multivariate logistic regression analysis was performed using independent variables that showed statistically significant differences in univariate analysis. However, independent variables with high Pearson correlation coefficients may have multicollinearity problems: BMI and waist circumference (r = 0.898), TC and Non-HDL-C (r = 0.945), TC and LDL-C (r = 0.873), LDL-C and Non-HDL-C (r = 0.863), and TG/HDL ratio and TG (r = 0.951), and TC/HDL ratio and non-HDL (r = 0.772). Additionally, non-HDL, LDL, TG/HDL ratio, and TC/HDL ratio variables were variables created using existing variables. Considering these points, the final variables were selected. The final variables were BMI, TC, TG, LDL-C, HDL-C, serum uric acid, and ALT.

As a result of the analysis, factors affecting dyslipidemia in adolescents were identified as TC, TG, LDL-C, and serum uric acid. In particular, as LDL-C and serum uric acid increased by 1 unit, the risk of developing dyslipidemia was 1.47 and 1.45 times higher, respectively (Table 4).

## 4. Discussion

This study was performed to compare the differences between the group with dyslipidemia and the group without dyslipidemia in Korean adolescents using data from the third year (2021) of the 8th KNHANES, and to identify significant factors affecting dyslipidemia adolescents.

In this study, the possibility of dyslipidemia in adolescents aged 12–18 was 28.1%, based on the weighted population, similar to the results reported in a study [1] using data from the 2007–2018 KNHANES. These results show that continued research is needed on health management policies and intervention strategies that can reduce the prevalence of dyslipidemia.

In this study, obesity, waist circumference, serum uric acid, and ALT were found to be significant factors affecting dyslipidemia in Korean adolescents aged 12–18. Physical activity 4–7 days a week was a significant factor affecting dyslipidemia only in male adolescents.

As a result of the analysis, the probability of dyslipidemia in adolescents increased 5.26 times for males and 7.76 times for females when the BMI percentile was in the range for ‘obesity’ compared to ‘underweight and normal’. This is similar to the reports in previous research on children and adolescents or adolescents only; the more obese they are, the higher the frequency of high-triglycerides [19]. Furthermore, as the degree of obesity increases, the probability of hypercholesterolemia also increases [20]. There were similar findings for high lipid levels and high frequencies of cardiovascular risk factors in overweight subjects [21,22] and higher prevalence of co-morbidities such as high-triglyceride and metabolic syndrome in obesity subjects [7]. However, abnormal trends in TC, LDL-C, and non-HDL-C levels were more pronounced in males with normal BMI than in overweight and obese males, with significant increases in normal BMI and overweight females, while not in obese females [23]. This shows that the prevalence of not only overweight and obese adolescents but also adolescents with normal body mass index is increasing in cases of abnormal blood lipid levels.

In addition, in this study, it was analyzed that an increase in waist circumference increased the possibility of dyslipidemia in both males and females. There are reports that waist circumference or waist-to-height ratio is useful in predicting cardiovascular disease risk factors in children [24,25], and studies on the relationship between waist circumference-to-height ratio and dyslipidemia need to be further investigated. Meanwhile, in this study, it was found that height was not significantly related to the prevalence of dyslipidemia. This is a different result from the report showing that short stature is related to an increase in the risk of dyslipidemia in Korean adults and adolescents [2], so further research on the relationship between height and dyslipidemia in adolescents is suggested.

In this study, when the serum uric acid level increased by 1 unit, the possibility of dyslipidemia prevalence increased by 1.56 times in males and 2.27 times in females, and the results of multivariate regression analysis also showed that serum uric acid was an important factor affecting dyslipidemia. Uric acid levels are high in obese children [26], and hyperuricemia is related to hypertension, metabolic syndrome, atherosclerosis, insulin resistance, type 2 diabetes, gouty arthritis, atrial fibrillation, and kidney stones [9,13,27,28,29,30]. Therefore, screening tests for these diseases are necessary for adolescents with increased uric acid levels.

Furthermore, according to this study, an increase in ALT level significantly increased the possibility of dyslipidemia in both male and female adolescents. These results are similar to previous research showing that children, adolescents, and adults with high ALT levels are more likely to have metabolic syndrome, dyslipidemia, hypertension, and diabetes than those with normal ALT levels, resulting in a higher cardiovascular risk [14,31,32,33]. With the recent increase in the probability of childhood obesity, the risk of non-alcoholic fatty liver disease is increasing, so continuous ALT and AST levels are required to monitor liver disease screening, especially in obese teenagers.

In this study, an increase in TG, LDL-C, and non-HDL-C levels increased the possibility of the prevalence of dyslipidemia, and an increase in HDL-C levels reduced the possibility of dyslipidemia, which was predicted in conjunction with the definition of dyslipidemia. According to a previous study, approximately 0.41% of Korean children and adolescents with dyslipidemia were subjects for lipid-lowering pharmacological treatment [34]. In this study, none of the adolescents with dyslipidemia were found to be taking the drug, so it is necessary to consider drug treatment according to the guidelines in parallel with lifestyle improvements for adolescents as well as adults. In addition, non-HDL-C is a reliable predictor of cardiovascular disease [35,36,37], does not require fasting for the test, is not affected by triglyceride concentration, contains arteriosclerotic lipoproteins including LDL-C, and is simple to calculate [38]. Therefore, the use of non-HDL-C is recommended as a screening method for dyslipidemia in children and adolescents [39]. In a previous study of adolescents, the average fasting plasma concentrations of TG, TC, HDL-C, LDL-C, and non-HDL-C were all higher in males than in females [40]. Further research is suggested to identify blood lipid levels and risk factors for dyslipidemia according to more diverse criteria such as age and BMI of adolescents, as well as sex.

The TG/HDL-C ratio and the TC/HDL-C ratio are known to be indicators of metabolic syndrome and cardiovascular risk with higher predictive power than individual lipid profiles in adolescents and adults [10,41]. In this study, the average TG/HDL-C ratio was 2.92 ± 0.15 in the dyslipidemia group and 1.26 ± 0.04 in the normal group. In addition, when the TG/HDL-C ratio increased by 1 unit, the possibility of dyslipidemia increased by 13.44 times for males and 6.81 times for females, and when the TC/HDL-C ratio increased by 1 unit the possibility of dyslipidemia increased by 19.91 times for males and 32.61 times for females. In previous research in children and adolescents, the average TG/HDL-C ratio was 1.77 in males and 1.72 in females, and the TG/HDL-C ratio tended to increase with age in males, whereas it tended to decrease with age in females [39].

In this study, FPG and HbA1c were not significantly related to the prevalence of dyslipidemia, but based on reports that the TG/HDL-C ratio in adults is related to insulin resistance, FPG, and HbA1c [11,42,43], it is necessary to look into the correlations between the TG/HDL-C ratio and insulin resistance, FPG, and HbA1c in adolescents as well.

In this study, male adolescents who engaged in physical activity 4–7 days a week were found to have 0.24 times reduced risk of developing dyslipidemia compared to those who did not engage in physical activity in the past 7 days. Metabolic syndrome diagnostic criteria include abdominal obesity, TG, HDL-C, blood pressure, and blood glucose, and the report that the possibility of prevalence of metabolic syndrome in adolescents increases by 1.054 times for every hour of sitting time per day [44] is similar to the results of this study. Additionally, it has been reported that light physical activity or resistance exercise and acute aerobic and high-intensity exercise improves blood lipid concentration and decreases the risk of cardiovascular disease in adults [45,46,47,48]. Therefore, further research is suggested to identify the relationship with dyslipidemia by considering adolescents’ sex and the type and intensity of physical activity, its duration, and the total amount of activity.

There was a significant difference in the percentage of adolescents with or without dyslipidemia depending on whether the mother was diagnosed with hyperlipidemia in this study. Unfortunately, the number of samples corresponding to the ‘yes’ of the mother’s diagnosis of hyperlipidemia was very small, so there was a limit to conducting an analysis to find that the variable was a significant influence factor of dyslipidemia. According to research related to family history, the odds ratio for dyslipidemia is significant at 1.868 in the group with a family history compared to the group without a family history of chronic diseases [49], and for every 1 unit increase in the mother’s TC, the adolescent’s TC increased by 0.23 mg/dL [8]. Therefore, it is necessary to find out in detail the relationship between the family history of the disease and adolescent dyslipidemia by securing the number of study subjects in family-history-related variables.

The following are this study’s limitations. First, since this study was a cross-sectional study using the 2021 KNHANES, the causal relationship between each variable and dyslipidemia could not be identified. Second, in relation to smoking and drinking experience, only dichotomous variables were used that classified subjects into smokers and non-smokers, and drinkers and non-drinkers, so the effects related to the amount of smoking and drinking could not be considered. Third, according to the KNHANES guidelines, pulse and blood pressure are measured on the right arm for standardization. However, because blood pressure was not measured in both arms, there may be differences in measured values. Fourth, due to the insufficient number of samples, there was a limit to conducting an analysis to find out whether the mother’s hyperlipidemia was a significant factor in adolescent dyslipidemia. Nevertheless, this study is significant in that it was conducted using data representing adolescents nationwide based on stratified cluster sampling design and complex sample design.

## 5. Conclusions

In conclusion, obesity, waist circumference, serum uric acid, and ALT are significant factors influencing dyslipidemia in adolescents. Physical activity 4–7 days a week was analyzed to be an important factor affecting dyslipidemia only in male adolescents. In particular, serum uric acid being an influencing factor of dyslipidemia in adolescents and the relationship between the TG/HDL-C ratio and the TC/HDL-C ratio and the possibility of dyslipidemia were the main contributions of this study.

The results of this study can be used as evidence for the risk assessment of adolescent dyslipidemia and the establishment of systematic health management guidelines according to risk factors for the prevention of adolescent dyslipidemia.

## Figures and Tables

**Table 1:** Comparison of demographic, lifestyle, psychological, and family history variables in the normal group and the dyslipidemia group (*n* = 381, *N* = 3,072,013).

Variables	Categories	Normal (*n* = 273, *N* = 2,208,197)	Dyslipidemia (*n* = 108, *N* = 863,816)	F * (*p*)
		*n* (%)	*n* (%)	
Sex	Male	140 (68.7)	61 (31.3)	1.48 (0.227)
	Female	133 (75.4)	47 (24.6)	
Age (years)	12–14	133 (71.9)	51 (28.1)	0.00 (0.990)
	15–18	140 (71.9)	57 (28.1)	
Household income ^†^	Low	24 (67.1)	14 (32.9)	0.70 (0.551)
	Middle low	65 (65.8)	27 (34.2)	
	Middle high	96 (76.8)	28 (23.2)	
	High	87 (72.7)	39 (27.3)	
Smoking experience	No	253 (70.7)	103 (29.3)	1.58 (0.211)
	Yes	20 (82.4)	5 (17.6)	
Drinking experience	No	224 (71.4)	91 (28.6)	0.07 (0.793)
	Yes	49 (73.4)	17 (26.6)	
Physical activity at least 60 min/day	None/last 7 days	179 (70.7)	73 (29.3)	2.57 (0.082)
	1–3 days/week	61 (67.4)	31 (32.6)	
	4–7 days/week	33 (88.6)	4 (11.4)	
Stress perception level	Much	62 (74.7)	26 (25.3)	1.06 (0.348)
	A little	156 (68.9)	67 (31.1)	
	Hardly	55 (78.1)	15 (21.9)	
Diagnosis of hyperlipidemia (father) ^†^	No	238 (71.0)	101 (29.0)	0.40 (0.640)
	Yes	28 (78.7)	5 (21.3)	
	Do not know/ No answer	6 (82.8)	2 (17.2)	
Diagnosis of hyperlipidemia (mother) ^†^	No	265 (73.4)	99 (26.6)	7.81 (0.001)
	Yes	0 (0.0)	8 (100.0)	
	Do not know/ No answer	7 (83.8)	1 (16.2)	

* Rao–Scott χ^2^ test; ^†^ excluding non-response; *n*, unweighted sample size; *N*, weighted sample size; %, weighted %.

**Table 2:** Differences in physiological and biochemical indicators when comparing the normal group and the dyslipidemia group (*n* = 381, *N* = 3,072,013).

Variables	Categories	Normal (*n* = 273, *N* = 2,208,197)	Dyslipidemia (*n* = 108, *N* = 863,816)	*t* or F * (*p*)
		*n* (%) or M ± SE	*n* (%) or M ± SE	
BMI percentile ^†^	Underweight/ Normal	210 (81.2)	54 (18.8)	20.41 (<0.001)
	Overweight	25 (62.3)	13 (37.7)	
	Obesity	35 (41.4)	41 (58.6)	
Height (cm) ^†^		165.22 ± 0.72	166.08 ± 1.01	−0.71 (0.480)
Waist circumference (cm) ^†^		71.18 ± 0.76	80.29 ± 2.00	−4.16 (<0.001)
SBP (mmHg) ^†^		107.25 ± 0.74	109.43 ± 1.03	−1.73 (0.085)
DBP (mmHg) ^†^		66.96 ± 0.75	67.45 ± 0.89	−0.45 (0.655)
TC (mg/dL)		154.98 ± 1.43	176.37 ± 4.10	−5.28 (<0.001)
TG (mg/dL)		65.11 ± 1.88	120.98 ± 5.41	−10.57 (<0.001)
LDL-C (mg/dL)		88.56 ± 1.28	108.41 ± 3.32	−5.83 (<0.001)
HDL-C (mg/dL)		53.40 ± 0.69	43.77 ± 1.35	6.11 (<0.001)
Non-HDL-C (mg/dL)		101.58 ± 1.42	132.61 ± 3.25	−9.38 (<0.001)
TG/HDL-C (mg/dL)		1.26 ± 0.04	2.92 ± 0.15	−10.93 (<0.001)
TC/HDL-C (mg/dL)		2.95 ± 0.04	4.13 ± 0.10	−10.97 (<0.001)
Hb (g/dL) ^†^		13.91 ± 0.09	14.02 ± 0.17	−0.55 (0.581)
Hct (%) ^†^		42.51 ± 0.24	42.90 ± 0.45	−0.75 (0.455)
FPG (mg/dL)		91.38 ± 0.52	92.17 ± 0.80	−0.83 (0.406)
HbA1C (%) ^†^		5.38 ± 0.02	5.42 ± 0.03	−1.01 (0.315)
Serum uric acid (mg/dL) ^†^		5.47 ± 0.11	6.41 ± 0.20	−4.04 (<0.001)
AST (IU/L)		19.82 ± 0.52	23.23 ± 1.79	−1.76 (0.079)
ALT (IU/L)		14.96 ± 0.77	24.12 ± 2.93	−2.98 (0.003)

* Rao–Scott χ^2^ test; ^†^ excluding non-response; *n*, unweighted sample size; *N*, weighted sample size; %, weighted %; BMI, body mass index; SBP, systolic blood pressure; DBP, diastolic blood pressure; TC, total cholesterol; TG, triglyceride; LDL-C, low-density lipoprotein-cholesterol; HDL-C, high-density lipoprotein-cholesterol; Hb, hemoglobin; Hct, hematocrit; FPG, fasting plasma glucose; HbA1c, hemoglobin A1c; AST, aspartate aminotransferase; ALT, alanine aminotransferase.

**Table 3:** Factors affecting dyslipidemia among Korean male and female adolescents (*n* = 381, *N* = 3,072,013).

Variables (Reference)	Categories	Male (*n* = 201, *N* = 1,624,503)		Female (*n* = 180, *N* = 1,447,510)	
		OR (95% CI)	*p*	OR (95% CI)	*p*
Age (12–14 years)	15–18 years	1.07 (0.54, 2.10)	0.847	0.91 (0.42, 1.96)	0.806
Household income (High) ^†^	Low	1.08 (0.28, 4.13)	0.912	1.62 (0.52, 5.07)	0.402
	Middle low	1.79 (0.64, 5.02)	0.267	0.93 (0.36, 2.41)	0.873
	Middle high	0.82 (0.31, 2.21)	0.696	0.77 (0.33, 1.82)	0.548
Smoking experience (No)	Yes	0.27 (0.07, 1.04)	0.056	1.27 (0.26, 6.27)	0.769
Drinking experience (No)	Yes	0.74 (0.25, 2.23)	0.590	1.12 (0.39, 3.26)	0.831
Physical activity at least 60 min/day (None)	1–3/week	1.08 (0.49, 2.37)	0.856	1.32 (0.55, 3.20)	0.536
	4–7/week	0.24 (0.06, 0.97)	0.045	0.46 (0.06, 3.27)	0.431
Stress perception level (Hardly)	Much	1.85 (0.59, 5.78)	0.291	0.91 (0.26, 3.23)	0.886
	A little	1.71 (0.75, 3.86)	0.198	1.49 (0.52, 4.28)	0.458
BMI percentile (Underweight/Normal) ^†^	Overweight	2.23 (0.77, 6.48)	0.138	3.02 (0.98, 9.35)	0.055
	Obesity	5.26 (2.51, 11.03)	<0.001	7.76 (2.94, 20.52)	<0.001
Height (cm) ^†^		1.00 (0.96, 1.04)	0.976	1.01 (0.94, 1.08)	0.891
Waist circumference (cm) ^†^		1.06 (1.03, 1.09)	<0.001	1.13 (1.06, 1.20)	<0.001
SBP (mmHg) ^†^		1.03 (1.00, 1.06)	0.092	1.01 (0.97, 1.05)	0.748
DBP (mmHg) ^†^		1.02 (0.98, 1.07)	0.315	0.98 (0.93, 1.03)	0.393
TC (mg/dL)		1.03 (1.01, 1.04)	<0.001	1.04 (1.02, 1.06)	<0.001
TG (mg/dL)		1.06 (1.04, 1.08)	<0.001	1.04 (1.03, 1.06)	<0.001
LDL-C (mg/dL)		1.03 (1.02, 1.05)	<0.001	1.04 (1.02, 1.06)	<0.001
HDL-C (mg/dL)		0.88 (0.82, 0.93)	<0.001	0.88 (0.81, 0.96)	0.002
Non-HDL-C (mg/dL)		1.05 (1.03, 1.07)	<0.001	1.07 (1.05, 1.09)	<0.001
TG/HDL-C (mg/dL)		13.44 (6.55, 27.57)	<0.001	6.81 (3.79, 12.25)	<0.001
TC/HDL-C (mg/dL)		19.91 (9.71, 40.84)	<0.001	32.61 (9.86, 107.88)	<0.001
Hb (g/dL) ^†^		1.14 (0.72, 1.81)	0.575	0.83 (0.59, 1.16)	0.278
Hct (%) ^†^		1.04 (0.88, 1.24)	0.639	0.95 (0.82, 1.09)	0.455
FPG (mg/dL)		1.02 (0.97, 1.07)	0.476	1.01 (0.95, 1.07)	0.764
HbA1C (%) ^†^		0.96 (0.24, 3.91)	0.959	3.06 (0.54, 17.26)	0.203
Serum uric acid (mg/dL) ^†^		1.56 (1.17, 2.06)	0.002	2.27 (1.54, 3.35)	<0.001
AST (IU/L)		1.02 (0.99, 1.05)	0.166	1.09 (0.98, 1.21)	0.116
ALT (IU/L)		1.03 (1.01, 1.05)	0.005	1.08 (1.02, 1.15)	0.009

^†^ Excluding non-response; *n*, unweighted sample size; *N*, weighted sample size; OR, odds ratio; CI, confidence interval; BMI, body mass index; SBP, systolic blood pressure; DBP, diastolic blood pressure; TC, total cholesterol; TG, triglyceride; LDL-C, low-density lipoprotein-cholesterol; HDL-C, high-density lipoprotein-cholesterol; Hb, hemoglobin; Hct, hematocrit; FPG, fasting plasma glucose; HbA1c, hemoglobin A1c; AST, aspartate aminotransferase; ALT, alanine aminotransferase.

**Table 4:** Factors affecting dyslipidemia among Korean adolescents (*n* = 381, *N* = 3,072,013).

Variables	Categories	Adjusted OR	95% CI	*p*
BMI percentile ^†^	Underweight/ Normal	1.00		
	Overweight	1.38	(0.23, 8.16)	0.721
	Obesity	0.54	(0.20, 1.50)	0.237
TC (mg/dL)		0.70	(0.54, 0.90)	0.005
TG (mg/dL)		1.08	(1.03, 1.15)	0.005
LDL-C (mg/dL)		1.47	(1.16, 1.88)	0.002
HDL-C (mg/dL)		1.00	(1.00, 1.00)	-
Serum uric acid (mg/dL) ^†^		1.45	(1.07, 1.97)	0.002
ALT (IU/L)		1.01	(0.99, 1.03)	0.255

^†^ Excluding non-response; OR, odds ratio; CI, confidence interval; BMI, body mass index; TC, total cholesterol; TG, triglyceride; LDL-C, low-density lipoprotein-cholesterol; HDL-C, high-density lipoprotein-cholesterol; ALT, alanine aminotransferase.

## Data Availability

Not applicable.

## References

[1] Jeong D.Y., Kim S.H., Seo M.Y., Kang S.Y., Park M.J. (2021). Trends in serum lipid profiles among Korean adolescents, 2007–2018. Diabetes Metab. Syndr. Obes..

[2] Oh N.K., Song Y.M., Kim S.H., Park M.J. (2019). Short stature is associated with increased risk of dyslipidemia in Korean adolescents and adults. Sci. Rep..

[3] McGill H.C., McMahan C.A., Zieske A.W., Malcom G.T., Tracy R.E., Strong J.P. (2001). Effects of nonlipid risk factors on atherosclerosis in youth with a favorable lipoprotein profile. Circulation.

[4] Miller J., Rosenbloom A., Silverstein J. (2004). Childhood obesity. J. Clin. Endocrinol. Metab..

[5] Lim J.S., Kim E.Y., Kim J.H., Yoo J.-H., Yi K.H., Chae H.W., Choi J.-H., Kim J.Y., Hwang I.T., The Committee of Dyslipidemia of Korean Children and Adolescents on behalf of Korean Society of Pediatric Endocrinology (KSPE) (2020). 2017 clinical practice guidelines for dyslipidemia of Korean children and adolescents. Ann. Pediatr. Endocrinol. Metab..

[6] National Heart, Lung and Blood Institute (2011). Expert panel on integrated guidelines for cardiovascular health and risk reduction in children and adolescents; summary report. Pediatrics.

[7] Lim H.J., Xue H., Wang Y. (2014). Association between obesity and metabolic co-morbidities among children and adolescents in South Korea based on national data. BMC Public Health.

[8] Nam J.H., Shin J., Jang S.-I., Kim J.H., Han K.-T., Lee J.K., Lim Y.J., Park E.-C. (2019). Associations between lipid profiles of adolescents and their mothers based on a nationwide health and nutrition survey in South Korea. BMJ Open.

[9] Byun J.S., Kim J.N., Song Y.S., Roh Y.K., Choi M.K. (2019). The relationship between hyperuricemia and triglyceride glucose index: Based on 2016 Korea National Health and Nutrition Examination Survey. Korean J. Fam. Pract..

[10] Chu S.Y., Jung J.H., Park M.J., Kim S.H. (2019). Risk assessment of metabolic syndrome in adolescents using the triglyceride/high-density lipoprotein cholesterol ratio and the total cholesterol/high-density lipoprotein cholesterol ratio. Ann. Pediatr. Endocrinol. Metab..

[11] Park J.M., Kang S.M., Lee J.W., Lee J.W., Kim S.W., Lee J.H., Cho J.B. (2015). Correlation between the ratio of triglyceride to high-density lipoprotein cholesterol and fasting glucose & hemoglobin A1C. Korean J. Fam. Pract..

[12] Jeong H.R., Shim Y.S., Lee H.S., Hwang J.S. (2021). Hemoglobin and hematocrit levels are positively associated with blood pressure in children and adolescents 10 to 18 years old. Sci. Rep..

[13] De Miranda J.A., Almeida G.G., Leão Martins R.I., Cunha M.B., Belo V.A., dos Santos J.E.T., Mourão-Júnior C.A., Lanna C.M.M. (2015). The role of uric acid in the insulin resistance in children and adolescents with obesity. Rev. Paul. Pediatr..

[14] Park H.K., Hwang J.S., Moon J.S., Lee J.A., Kim D.H., Lim J.S. (2013). Healthy range of serum alanine aminotransferase and its predictive power for cardiovascular risk in children and adolescents. J. Pediatr. Gastroenterol. Nutr..

[15] Park S.H., Park H.Y., Kang J.W., Park J.S., Shin K.J. (2012). Aminotransferase upper reference limits and the prevalence of elevated aminotransferases in the Korean adolescent population. J. Pediatr. Gastroenterol. Nutr..

[16] Korea Disease Control and Prevention Agency Guidelines for the Use of Raw Data from the 8th (2019–2021) Korea National Health and Nutrition Examination Survey (KNHANES); Report No. 117002. https://knhanes.kdca.go.kr/knhanes/sub03/sub03_02_05.do.

[17] Roberts W.C. (1988). The Friedewald-Levy-Fredrickson formula for calculating low-density lipoprotein cholesterol, the basis for lipid-lowering therapy. Am. J. Cardiol..

[18] Kim J.H., Yun S., Hwang S.-S., Shim J.O., Chae H.W., Lee Y.J., Lee J.H., Kim S.C., Lim D., Yang S.W. (2018). The 2017 Korean national growth charts for children and adolescents: Development, improvement, and prospects. Korean J. Pediatr..

[19] Sung T.J., Kim D.H., Hong Y.J., Son B.K., Chang K.J., Park J.Y., Kim S.K. (2003). Effective screening test for obesity in obese adolescents and the correlation among obesity index, body mass index and serum lipid profile. J. Korean Pediatr. Soc..

[20] Nho H.N., Kim C.R., Uhm J.H., Kim J.T., Jin S.M., Seo J.Y., Hahn H.W., Park H.Y., Yoon H.S., Ahn Y.M. (2009). The prevalence of obesity and metabolic abnormalities in Korean pediatric population. Korean J. Pediatr. Gastroenterol. Nutr..

[21] Freedman D.S., Dietz W.H., Srinivasan S.R., Berenson G.S. (1999). The relation of overweight to cardiovascular risk factors among children and adolescents: The Bogalusa Heart Study. Pediatrics.

[22] Reinehr T., Andler W., Denzer C., Siegried W., Mayer H., Wabitsch M. (2005). Cardiovascular risk factors in overweight German children and adolescents: Relation to gender, age and degree of overweight. Nutr. Metab. Cardiovasc. Dis..

[23] Song K., Jeon S., Lee H.S., Choi H.S., Suh J., Kwon A., Kim H.-S., Chae H.W. (2021). Trends of dyslipidemia in Korean youth according to sex and body mass index: Based on the Korea National Health and Nutrition Examination Survey (2007–2018). J. Pediatr..

[24] Lee G.E., Choo J.N. (2017). Childhood obesity and cardiovascular health: Using 2010–2012 data of the Korea National Health and Nutrition Examination Survey. Korean J. Health Promot..

[25] Savva S., Tornaritis M., Savva M., Kourides Y., Panagi A., Silikiotou N., Georgiou C., Kafatos A. (2000). Waist circumference and waist-to-height ratio are better predictors of cardiovascular disease risk factors in children than body mass index. Int. J. Obes. Relat. Metab. Disord..

[26] Gil-Campos M., Aguilera C.M., Cañete R., Gil A. (2009). Uric acid is associated with features of insulin resistance syndrome in obese children at prepubertal stage. Nutr. Hosp..

[27] Cardoso A.S., Gonzaga N.C., Medeiros C.C., Carvalho D.F. (2013). Association of uric acid levels with components of metabolic syndrome and non-alcoholic fatty liver disease in overweight or obese children and adolescents. J. Pediatr..

[28] Viazzi F., Antolini L., Giussani M., Brambilla P., Galbiati S., Mastriani S., Stella A., Pontremoli R., Valsecchi M.G., Genovesi S. (2013). Serum uric acid and blood pressure in children at cardiovascular risk. Pediatrics.

[29] Civantos Modino S., Guijarro de Armas M.G., Monereo Mejías S., Montaño Martínez J.M., Iglesias Bolaños P., Merino Viveros M., Ladero Quesada J.M. (2012). Hyperuricemia and metabolic syndrome in children with overweight and obesity. Endocrinol. Nutr..

[30] Pacifico L., Cantisani V., Anania C., Bonaiuto E., Martino F., Pascone R., Chiesa C. (2009). Serum uric acid and its association with metabolic syndrome and carotid atherosclerosis in obese children. Eur. J. Endocrinol..

[31] Cho N.H., Jang H.C., Choi S.H., Kim H.R., Lee H.K., Chan J.C., Lim S. (2007). Abnormal liver function test predicts type 2 diabetes: A community-based prospective study. Diabetes Care.

[32] Patel D.A., Srinivasan S.R., Xu J.H., Chen W., Berenson G.S. (2007). Persistent elevation of liver function enzymes within the reference range is associated with increased cardiovascular risk in young adults: The Bogalusa Heart Study. Metabolism.

[33] Yun K.E., Shin C.Y., Yoon Y.S., Park H.S. (2009). Elevated alanine aminotransferase levels predict mortality from cardiovascular disease and diabetes in Koreans. Atherosclerosis.

[34] Yang S., Hwang J.S., Park H.K., Lee H.S., Kim H.S., Kim E.Y., Lim J.S. (2012). Serum lipid concentrations, prevalence of dyslipidemia, and percentage eligible for pharmacological treatment of Korean children and adolescents; data from the Korea National Health and Nutrition Examination Survey IV (2007–2009). PLoS ONE.

[35] Moriyama K., Takahashi E. (2016). Non-HDL cholesterol is a more superior predictor of small-dense LDL cholesterol than LDL cholesterol in Japanese subjects with TG levels < 400 mg/dL. J. Atheroscler. Thromb..

[36] Liu A., Reaven G.M. (2013). Is measurement of non-HDL cholesterol an effective way to identify the metabolic syndrome?. Nutr. Metab. Cardiovasc. Dis..

[37] Blaha M.J., Blumenthal R.S., Brinton E.A., Jacobson T.A. (2008). National Lipid Association Taskforce on Non-HDL Cholesterol: The importance of non-HDL cholesterol reporting in lipid management. J. Clin. Lipidol..

[38] Virani S.S. (2011). Non-HDL cholesterol as a metric of good quality of care: Opportunities and challenges. Tex. Heart Inst. J..

[39] Shim Y.S., Baek J.W., Kang M.J., Oh Y.J., Yang S., Hwang I.T. (2016). Reference values for the triglyceride to high-density lipoprotein cholesterol ratio and non-high-density lipoprotein cholesterol in Korean children and adolescents: The Korea National Health and Nutrition Examination Surveys 2007–2013. J. Atheroscler. Thromb..

[40] Kwon S.Y., Na Y.A. (2018). Distribution of the fasting lipid levels and validation of the reference interval in Korean adolescents. Korean J. Clin. Lab. Sci..

[41] Kimm H., Lee S.W., Lee H.S., Shim K.W., Cho C.Y., Yun J.E., Jee S.H. (2010). Associations between lipid measures and metabolic syndrome, insulin resistance and adiponectin.-Usefulness of lipid ratios in Korean men and women-. Circ. J..

[42] McLaughlin T., Abbasi F., Cheal K., Chu J., Lamendola C., Reaven G. (2003). Use of metabolic markers to identify overweight individuals who are insulin resistant. Ann. Intern. Med..

[43] Bovet P., Faeh D., Gabriel A., Tappy L. (2006). The prediction of insulin resistance with serum triglyceride and high-density lipoprotein cholesterol levels in an East African population. Arch. Intern. Med..

[44] Moon W.J. (2019). The relation among adolescents metabolic syndrome, dietary life, physical activity and mental health-Using 7th national nutrition survey of 1st year (2016)-. J. Korea Acad. Ind. Coop. Soc..

[45] Jeong J.H., Choi Y.S., Kim S.M., Choi S.Y., Kim J.E., Kim E.Y., Lee H.Y., Jung J.H., Jin R. (2014). Correlation between physical activity status and dyslipidemia in Korean adults: The 2010 Korea National Health and Nutrition Examination Survey. Korean J. Clin. Geriatr..

[46] Lira F.S., Zanchi N.E., Lima-Silva A.E., Pires F.O., Bertuzzi R.C., Santos R.V., Caperuto E.C., Kiss M.A., Seelaender M. (2009). Acute high-intensity exercise with low energy expenditure reduced LDL-C and total cholesterol in men. Eur. J. Appl. Physiol..

[47] Park D.H., Ransone J.W. (2003). Effects of submaximal exercise on high-density lipoprotein-cholesterol subfractions. Int. J. Sports Med..

[48] Magkos F., Tsekouras Y.E., Prentzas K.I., Basioukas K.N., Matsama S.G., Yanni A.E., Kavouras S.A., Sidossis L.S. (2008). Acute exercise-induced changes in basal VLDL-triglyceride kinetics leading to hypotriglyceridemia manifest more readily after resistance than endurance exercise. J. Appl. Physiol..

[49] Lee N.C., Kim M.J., Choi H.J., Lee J.S., Jung D. (2021). A study on the prevalence and risk of family history for chronic diseases: Findings from the Korea National Health and Nutrition Examination Survey 2019. J. Converg. Inf. Technol..

